# Improvement of Bone Health and Initiation of Puberty Development in Camurati-Engelmann Disease With Glucocorticoid and Losartan Treatment: A Case Report and Review of Literature

**DOI:** 10.3389/fendo.2022.882144

**Published:** 2022-06-17

**Authors:** Lijia Cui, Qian Li, Wenmin Guan, Wei Yu, Xiang Li, Weibo Xia, Yan Jiang

**Affiliations:** ^1^ Department of Endocrinology, Peking Union Medical College Hospital, Chinese Academy of Medical Science, Beijing, China; ^2^ Department of Radiology, Peking Union Medical College Hospital, Chinese Academy of Medical Science, Beijing, China

**Keywords:** progressive diaphyseal dysplasia, hypogonadotropic hypogonadism, prednisone, angiotensin receptor II blocker, bone mass

## Abstract

Camurati-Engelmann Disease (CED) is a rare sclerosing bone disease, sometimes associated delayed puberty. The treatment effect of glucocorticoid and angiotensin II receptor blocker (ARB) in bone health and puberty development remain unclear. We report a case of an 18-year-old girl who presented for a history of an enlarged head, pain of lower limbs, and no menstrual onset or breast development. Radiographs revealed thickening of skull and cortices in the diaphysis but sparse bone trabeculae in the spine and metaphysis. Sanger sequencing detected a mutation of c. 652C>T (p. R218C) in the gene *TGFB1* and confirmed the diagnosis of CED. After treatment of a medium-to-small dosage of prednisone and losartan for 28 months, we observed improvement of bone mass in spine and hip and body fat mass and found initiation of puberty development. By a systemic review of current treatment strategies in patients with CED, we found that most cases reported relief of bone pain with treatment of glucocorticoid or ARB, but none has reported the outcome of hypogonadotropic hypogonadism. We propose that long-term use of glucocorticoid combined with ARB may inhibit the activation of TGFβ1 in CED, improve adipogenesis, and thus initiate puberty development and improve the bone mass in spine and hip.

## Introduction

Camurati-Engelmann Disease (CED), also called progressive diaphyseal dysplasia, is a rare autosomal dominant sclerosing bone disease, led by activating mutations in the Transforming Growth Factor Beta 1 (*TGFB1*) gene. More than 300 cases of CED have been reported up to date (1), with an estimated prevalence of CED of 1 in 1,000,000 (2). The radiographs of CED are characterized by thickening of skull and diaphysis of long tubular bones, whereas the spine and metaphysis of lone bones are spared. Bone pain and muscle weakness are typical manifestations in patients with CED (1), and delayed puberty has been commonly reported (2–4).

There were limited experiences for treating CED, due to the rarity of the disease. Some researchers reported the effect of glucocorticoid in relieving bone pain and improving muscle weakness (5). However, researchers are concerned that long-term use of glucocorticoid may worsen osteoporosis. Angiotensin II receptor blocker (ARB) has also been reported to relieve bone pain and improve lean and adipose tissue in two CED cases (6, 7). However, the effect of combined use of glucocorticoid and ARB in patients with CED on bone health and puberty development is currently unknown.

Here, we report on an 18-year-old female patient of CED. After treatment of a medium-to-small dosage of prednisone combined with losartan for 28 months, we observed improvement of bone mass in the spine and hip and body fat mass and found initiation of puberty development. A literature review was performed to summarize the effect of current treatment strategies on patients with CED.

## Patient and Methods

### Patient

An 18-year-old girl presented to our clinic. The patient had a history of enlarged head and headache since the age of 6 and had been experiencing pain of the lower limbs since the age of 7. She experienced hearing loss since the age of 16. She had no menstrual onset and no breast development until admission. Her parents and brother (aged 7 years old) developed normally. We obtained written informed consent from the patient and her family. All clinical and genetic studies were approved by ethical review committee of Peking Union Medical College Hospital.

Assessment of bone turnover markers, hematology and liver and renal function, whole-body dual X-ray energy absorptiometry scan (DXA), X-ray, and bone scintigraphy were performed on the patient at the first visit and during the follow-up. Body mass index (BMI) was calculated as weight/height^2^. Both fat mass and muscle mass were measured by whole-body DXA. Fat mass index (FMI) was calculated as fat mass/height^2^. For females, FMI < 3.5 kg/m^2^ was defined as extremely fat deficient; 3.5 ≤ FMI < 4 kg/m^2^ was defined as moderately fat deficient; 4 ≤ FMI < 5 kg/m^2^ was defined as mildly fat deficient; and 5 ≤ FMI < 9 kg/m^2^ was defined as normal (8). Skeletal muscle index (SMI) was calculated as muscle mass/height^2^. For female, SMI <5.45kg/m^2^ was defined as sarcopenia (9, 10).

A gonadotropin-releasing hormone (GnRH) stimulation test was performed to evaluate the hypothalamus–pituitary–gonad (HPG) axis in the patient. The test was performed by administering triptorelin acetate 0.1 mg intramuscularly and testing luteinizing hormone (LH) and follicle-stimulating hormone (FSH) 60 min later. For females, LH < 6 U/L indicated no puberty development or hypogonadotropic hypogonadism (HH); 6 ≤ LH ≤ 18 U/L indicated puberty development initiated or partial HH; and LH > 18 U/L indicated the HPG axis in adult (11).

Genomic DNA was extracted from peripheral blood leukocytes using standard methods of QIAampDNA blood mini kit (QIAGEN, German). All seven exons, 5′UTR, and 3′untranslated regions (UTR) in *TGFB1* gene were PCR-amplified for the patient and her parents and brother and sequenced with an ABI 3730 DNA Analyzer (Applied Biosystems, Foster City, CA). Sequences were compared to the *TGFB1* gene (NM_000660) to locate the mutation site.

### Literature Review on the Treatment of CED

A literature review was performed regarding the treatment of CED in NCBI, up to September 7, 2021, searched by keywords of [camurati engelmann disease (Title)] OR [progressive diaphyseal dysplasia (Title)].

## Result

### Case Report

**Figure 1:**
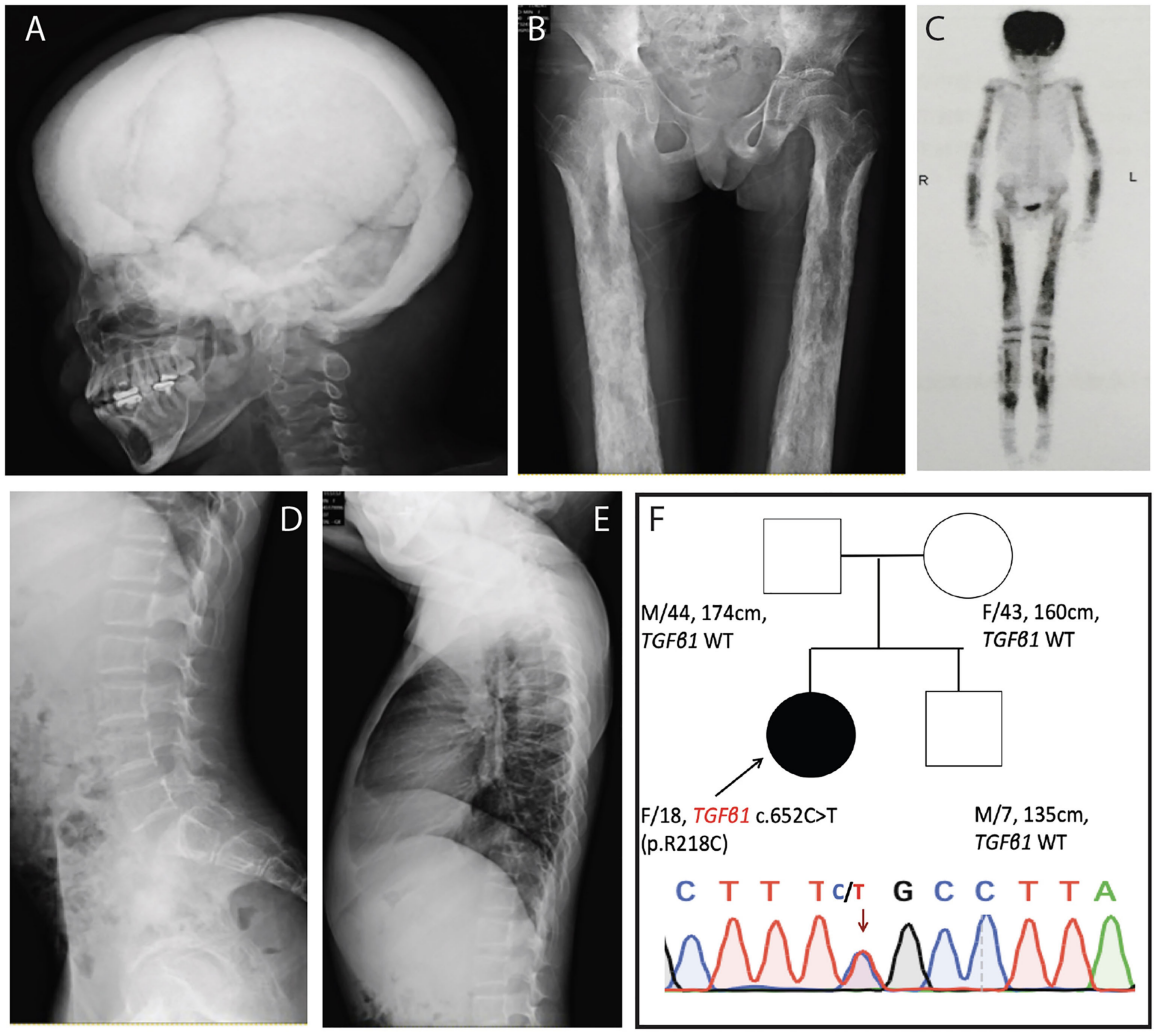
Clinical manifestation of the patient with CED. **(A, B)** X-ray revealed thickening of skull and thickening of cortices in the diaphysis of both femur. **(C)** Bone scintigraphy revealed high uptake in skull and the diaphysis of upper and lower limbs. **(D, E)** X-ray revealed sparse bone trabeculae in thoracic and lumbar spine. **(F)** Sanger sequencing detected a mutation of c.652C>T (p. R218C) in the gene *TGFB1* (NM_000660) in the patient, but neither in her parents nor her brother.

On physical examination, the patient was thin, with a height of 155 cm (10th–25th), weight of 41 kg (3rd–10th), and BMI of 17.07 kg/m^2^. Blood pressure was 147/95 mmHg. Her skull was enlarged, and her long bones were thickened. The breast and public hair were both in Tanner stage 1. Laboratory results were shown in **Table 1**. The X-ray revealed (**Figure 1**) thickening of the skull and thickening of cortices in the diaphysis but sparse bone trabeculae in the spine. Bone scintigraphy (**Figure 1C**) revealed high uptake of skull and diaphysis of long bones. Sanger sequencing detected a mutation of c. 652C>T (p. R218C) in the gene *TGFB1* (NM_000660) in the patient, but not in her parents or her brother. R218C is the hotspot mutation site in *TGFB1* and accounted for nearly 60% of mutations in patients with CED (12). On the basis of clinical manifestations and molecular analysis, the diagnosis of CED in the patient was confirmed.

**Table 1:** Demographic features of the patient with CED during 28-month follow-up.

	At presentation	7 months	18 months	28 months
**Treatment**				
Prednisone	30 mg qd × 10 days →20 mg qd × 10 days →10 mg qd × 2 weeks	7.5 mg qd × 1 month →5 mg qd × 1 month →3.75 mg qd × 3 months →5 mg qd × 1 month	10 mg qd × 11 month	10 mg qd × 2 months →12.5 mg qd × 5 months →15 mg qd × 3 months
Losartan	50mg qd × 28 months			
Calcium and Vitamin D	Vitamin D2 200,000 IU intramuscular injection once → Calcium carbonate 500 mg tid + Vitamin D3 1,200 IU qd × 28 months			
**Laboratory results**				
ESR mm/h	64	44	39	26
PTH pg/ml	83.7	22.8	24.2	–
25OHD ng/ml	6.7	27.8	41.1	39.6
Calcium mmol/L	2.27	2.37	2.40	2.38
Phosphate mmol/L	1.49	1.82	1.38	1.31
ALP U/L	387	337	413	311
βCTX ng/ml	4.56	2.76	3.36	2.83
P1NP ng/ml	1627	902	1044	447
LH U/L	<0.2	6.33	4.55	–
FSH U/L	1.38	7.41	1.65	–
Estradiol pg/ml	32	68	43	–
LH in GnRH stimulation test U/L	8.15	–	36.4	–
**Bone, muscle, and fat mass measurements**				
BMD Lumbar spine L1–L4	0.577 g/cm^2^ Z-score -4.7	–	0.644 g/cm^2^ Z-score -3.1	0.636 g/cm^2^ Z-score -3.5
BMD Femoral neck	0.498 g/cm^2^ Z-score -3.5	–	0.773 g/cm^2^ Z-score -0.9	0.631 g/cm^2^ Z-score -2.2
BMD Total hip	0.819 g/cm^2^ Z-score -1.0		0.881 g/cm^2^ Z-score -0.2	0.832 g/cm^2^ Z-score -0.7
BMI kg/m^2^	17.1	–	16.7	18.1
FMI kg/m^2^	1.51	–	2.16	2.06
SMI kg/m^2^	5.83	–	5.70	5.72
**Puberty measurements**				
Tanner stage	Breast 1 Pubic hair 1	Breast 2 Pubic hair 1	Breast 3 Pubic hair 2	Breast 4 Pubic hair 3
Menstrual onset	No menstrual onset			
Uterine size in ultrasound	2.1 × 1.7 × 0.8 cm	–	–	3.6 × 3.5 × 2.4 cm
Ovary size in ultrasound	1.9 × 1.2 cm (L) 2.1 × 1.3 cm (R)			2.1 × 1.9 cm (L) 3.2 × 1.8 cm (R)
Bone age	11–12 years	–	13–14 years	14–16 years

ESR, erythrocyte sedimentation rate; PTH, parathyroid hormone; 25OHD, 25 hydroxyl vitamin D; βCTX, C-terminal cross-linked telopeptide of type I collagen; P1NP, procollagen type I intact N-terminal propeptide; LH, luteinizing hormone; FSH, follicle-stimulating hormone; BMI, body mass index; FMI, fat mass index; SMI, skeletal muscle index.

**Figure 2:**
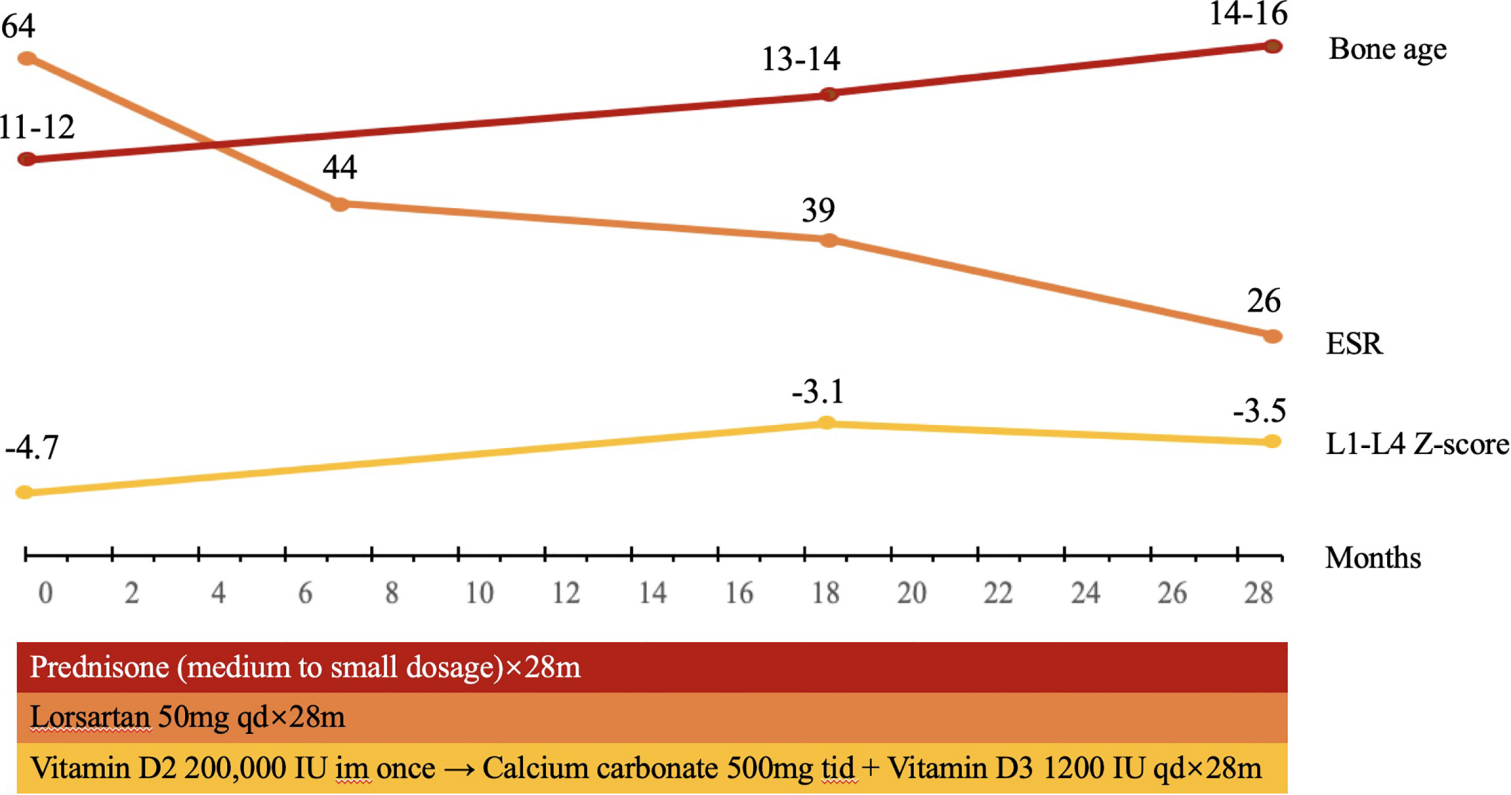
The timeline of treatment for the patient with CED. The patient was treated with prednisone, initiated at 30mg/day, decreased to 10 mg/day in 20 days, and then adjusted between 5 and 15 mg/day. Losartan 50 mg/day was given to the patient together with prednisone. Vitamin D2 of 200,000 IU intramuscular injection was given once, continued with calcium carbonate of 500 mg tid and vitamin D3 1200 IU qd. During the follow-up for 28 months, the BMD in L1–L4 in the patient significantly increased. Bone age increased from 11–12 years to 14–16 years, and Tanner stage of pubic hair and breast of the patient increased from stage 1 to 3 and from stage 1 to 4, which indicated that puberty was initiated and developed.

Treatment of prednisone was initiated at 30 mg/day and was decreased to 10mg/day in 20 days (**Table 1** and **Figure 2**). The dose of prednisone was then adjusted between 5 and 15 mg/day during the 28-months follow-up. Losartan (50 mg/day) was given to the patient together with prednisone. Because the patient had severe vitamin D deficiency at presentation, vitamin D2 of 200,000 IU intramuscular injection was given. Calcium carbonate of 500 mg tid and vitamin D3 of 1,200 IU qd were continued to the patient. The patient was followed up in clinic for 28 months, with physical examination, whole-body DXA, and assessment of bone turnover markers, hematology, and liver and renal function.

### Bone, Muscle, and Fat Mass Measurements

The bone mineral density (BMD) in the lumbar spine and femoral neck was 0.577 g/cm^2^ and 0.498 g/cm^2^ before treatment. After treatment of a medium-to-small dosage of glucocorticoid and losartan, the BMD in L1–L4 increased to 0.636 g/cm^2^, and the BMD in the femoral neck increased to 0.631 g/cm^2^ (**Table 1**). The FMI was 1.51 kg/m^2^ before treatment, which indicated extreme fat deficiency, and increased to 2.16 kg/m^2^ after treatment. The skeletal mass index (SMI) was 5.83 kg/m^2^, excluding the diagnosis of sarcopenia, and did not change significantly after treatment (**Table 1**).

### Puberty Development

The patient presented no menstrual onset or breast development at the age of 18. Laboratory results revealed serum LH < 0.2I U/L, FSH of 1.48 IU/L, estradiol of 23 pg/ml, progesterone of 0.32 ng/ml Sixty minutes after triptorelin acetate was administered, serum LH increased to 8.15 IU/L. Magnetic resonance imaging (MRI) revealed normal morphology of the pituitary. After treatment of a medium-to-small dosage of prednisone and losartan for 28 months, Tanner stage of pubic hair and breast of the patient increased from stage 1 to 3 and from stage 1 to 4, respectively. Serum LH after GnRH stimulation increased to 36.4 IU/L. Bone age also increased from 11–12 years to 14–16 years. The uterine size increased from 2.1 × 1.7 × 0.8 cm to 3.6 × 3.5 × 2.4 cm (**Table 1**). All the above findings indicated that puberty was initiated and developed with the treatment of prednisone and losartan.

### Literature Review on the Treatment of CED

A total of 180 literature studies were found from National Center of Biotechnology Information (NCBI), up to September 7, 2021, searched by keywords of (camurati engelmann disease[Title]) OR (progressive diaphyseal dysplasia[Title]). Ten literature studies presenting 30 CED cases with clear treatment strategies and long-term follow-up were kept for final analysis (**Table 2**). The most common strategy in CED is glucocorticoid. Prednisone was always started from a medium-to-large dosage and decreased to a small dosage for maintenance. The treatment course varies from 0.5 to 10 years. ARB has been used in two cases, with a treatment course of 1.5–3.2 years. All patients with glucocorticoid or ARB treatment reported bone pain relief and some have muscle weakness improved. Bisphosphonates, calcitonin, the receptor activator of nuclear factor-kappa B ligand (RANKL) monoantibodies, and tumor necrosis factor α (TNFα) monoantibodies have also been reported. However, among those 30 CED cases, none has reported the treatment effect of hypogonadotropic hypogonadism or puberty development.

**Table 2:** Summary of case reports on the treatment effect on bone and hypogonadism in patients with CED.

	Gender/Age	Treatment	Effect on bone	Effect on hypogonadism	Effect on muscle or fat	Ref.
**Bisphosphonates**						
1	F/20	Alendronate 5 mg qd × 2 years	Bone pain relieved, lumbar and femoral neck BMD improved	NA	NA	(13)
2	M/23	Alendronate 5 mg qd ×6 months	Bone pain relieved	NA	NA	(13)
3	F/27	Pamidronate 60 mg qow ×10 weeks	Bone pain worse	NA	NA	(14)
4	F/43	Zoledronate 5 mg once	Bone pain worse	NA	NA	(15)
5	F/31	Alendronate 5 mg qd × 6 months → clodronate 1,800 mg iv once	Bone pain worse	NA	NA	(16)
6	F/24	Risedronate 10 mg qd × 2 months → alendronate 40 mg qd × 2 months → clodronate 600 mg qd × 3 days	Bone pain worse	NA	NA	(16)
7	F/19	Zoledronate 0.02 mg/kg every 4 months × 2years	Bone pain relieved, lumbar and femoral neck BMD improved	NA	NA	(17)
8	M/7	Neridronate 1 mg/kg every 4 months ×16 monts → zoledronate 0.015 mg/kg every 4–6 months × 18 months	Bone pain relieved, femoral neck BMD improved	NA	NA	(17)
**Glucocorticoid**						
9	F/27	Dexamethasone 5 mg qd × 2 weeks	Bone pain relieved	NA	NA	(14)
10	F/19	Prednisone 0.3 mg/kg/d → 0.2 mg/kg/day × 10 years	Bone pain relieved	NA	NA	(18)
11	F/17	Prednisone 0.3 mg/kg/day → 0.2 mg/kg/day × 2 years	Bone pain relieved	NA	Muscle weakness improved	(18)
12	M/40	Prednisone 0.7 mg/kg/day → 0.4 mg/kg/day × 2 years	Bone pain relieved, hyperostosis of diaphysis remained	NA	Muscle weakness improved	(18)
13	F/38	Prednisone 0.5 mg/kg/day → 0.3 mg/kg/day × 2 years	Bone pain relieved	NA	Muscle weakness improved	(18)
14	M/36	Prednisone 0.6 mg/kg/day → 0.5 mg/kg/day × 1.5 years	Bone pain relieved, hyperostosis of diaphysis remained	NA	Muscle weakness improved	(18)
15	M/2.3	Prednisone 0.9 mg/kg/day → 0.3 mg/kg/day × 2.5 years	Bone pain relieved, hyperostosis of diaphysis remained	NA	Muscle weakness improved	(18)
16	F/13	Prednisone 0.5 mg/kg/day → 0.3 mg/kg/day × 1 year	Bone pain relieved	NA	NA	(18)
17	M/11	Prednisone 0.5 mg/kg/day → 0.3 mg/kg/day × 3 years	Bone pain relieved, hyperostosis of diaphysis remained	NA	Muscle weakness improved	(18)
18	M/5.5	Prednisone 0.6 mg/kg/day → 0.3 mg/kg/day × 3 years	Bone pain relieved, hyperostosis of diaphysis remained	NA	NA	(18)
19	F/10	Prednisone 0.5 mg/kg/day → 0.2 mg/kg/day × 2 years	Bone pain relieved	NA	NA	(18)
20	F/7	Prednisone 0.5 mg/kg/day → 0.25 mg/kg/day × 10 years	Bone pain relieved, hyperostosis of diaphysis remained	NA	Muscle weakness improved	(18)
21	M/10.8	Prednisone 0.8 mg/kg/day → 0.4 mg/kg/day × 0.5 years	Bone pain relieved, hyperostosis of diaphysis remained	NA	Muscle weakness improved	(18)
**ARB**						
22	M/13	Losartan 50 mg qd × 1.5 years	Bone pain relieved	NA	Muscle weakness improved	(7)
23	F/9	Losartan 0.75 mg/kg/day × 12 weeks → 1 mg/kg/day × 3.2 years	Bone pain relieved, lumbar spine BMD improved	NA	Muscle weakness improved, fat and lean mass improved	(6)
**Glucocorticoid+ARB**						
24	M/21	Deflazacort 1–1.2 mg/kg/day × 1–3 monts Losartan 0.8–1.2 mg/kg/day × 2 years	Bone pain relieved, BMD improved	NA	Muscle weakness improved	(19)
25	M/36		Bone pain relieved	NA	Muscle weakness improved	(19)
26	F/46		Bone pain relieved	NA	Muscle weakness improved	(19)
27	M/11		Bone pain relieved	NA	Muscle weakness improved	(19)
**Calcitonin**						
28	M/25	Calcitonin 200IU/day × 3months	Bone pain relieved	NA	NA	(20)
**RANKL monoantibody**						
29	F/66	Denosumab every 6 months × 6 months	Bone pain relieved, lumbar spine and total hip BMD improved	NA	NA	(21)
**TNFα monoantibody**						
30	F/46	Infliximab 5 mg/kg every 8 weeks × 2 years	Bone pain relieved, Lumbar BMD improved, femoral neck BMD decreased, hyperostosis of diaphysis remained	NA	NA	(22)

NA, Not available.

## Discussion

In this study, we report on an 18-year-old female patient of CED, presenting thickened skull and cortices in diaphysis, osteoporosis in spine and hip, and hypogonadotropic hypogonadism. With the treatment of a small-to-medium dosage of prednisone and losartan for 28 months, we observed improvement of bone mass in the spine and hip and body fat mass, and initiation of puberty development.

### Mechanism of Thickened Skull and Cortices of Diaphysis But Low BMD in the Spine and Femoral Neck in CED

**Figure 3:**
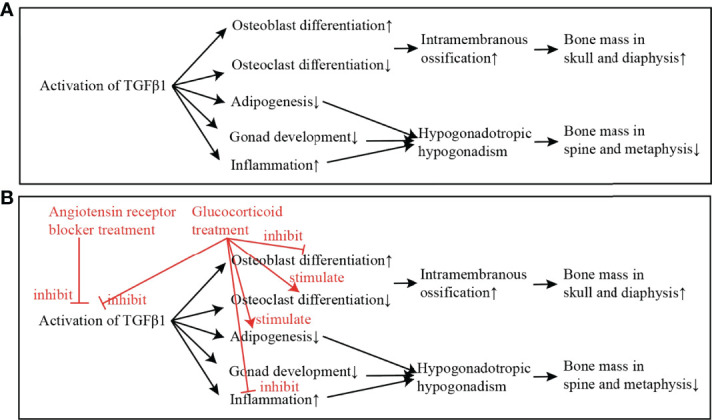
The mechanism of the activation of TGFβ1 in causing CED, and the putative effects of glucocorticoid and angiotensin receptor blocker (ARB) in improving the low bone mass and hypogonadism in CED. **(A)** The mechanism of TGFβ1 in causing CED: The activation of TGFβ1 in CED increases the differentiation of osteoblast and decreases the differentiation of osteoclast. Intramembrane ossification, which is regulated by osteoblast and osteoclast, plays a major role in the formation of skull and diaphysis. This may explain why patients with CED present increase of bone mass only in skull and diaphysis of long bones. On the other hand, activated TGFβ1 inhibits adipogenesis, inhibits gonad development, and increases inflammation. These effects lead to hypogonadotropic hypogonadism in CED, which may attribute to the low bone mass in spine and metaphysis. **(B)** Treatment of ARB directly inhibits the activation of TGFβ1; and the treatment of glucocorticoid inhibits the activation of TGFβ1 and also stimulates adipogenesis, and thus, the adipose mass is increased and puberty development is gradually initiated.

Typical clinical manifestations of CED include an increase of bone mass in the skull and diaphysis of long bones. The activation of TGFβ1 has been found to increase the differentiation of osteoblast and decrease the differentiation of osteoclast (23). Intramembrane ossification, which is regulated by osteoblast and osteoclast, plays a major role in the formation of the skull and the cortex of diaphysis. This may explain why patients with CED present an increase of bone mass only in the skull and diaphysis of long bones (**Figure 3A**).

On the other hand, low BMD in spine and femoral neck was found in the patient with CED. Estrogen exerts protective effects on bone (24), and the Tanner stage was the major determinant of BMD in girls (25). Previous studies have also reported that patients with hypogonadotropic hypogonadism had lower BMD compared with normal controls (26). We therefore proposed that low BMD in the lumbar spine and femoral neck in our patient with CED was mostly attributed to the hypogonadism.

### Mechanism of Lack of Fat Mass in CED

This patient presented a severe lack of fat mass and normal lean mass. Most cases of CED reported muscle weakness, but few of them have had fat mass and lean mass measured before and during treatment (**Table 2**). Adipogenesis is a process when fibroblasts develop into adipocytes. Several stimulators and inhibitors are involved in adipogenesis, among which TGFβ1 signaling pathway is a well-established pathway that inhibits adipogenesis (27). Therefore, we proposed that the lack of fat mass in patient with CED may be attributed to the over activation of TGFβ1.

### Mechanism of Hypogonadotropic Hypogonadism in CED

Patients with CED always present hypogonadotropic hypogonadism (2, 3). The reason for hypogonadotropic hypogonadism in CED has been proposed as follows (**Figure 3A**) 1) Adipose atrophy inhibits the secretion of GnRH and the release of LH and FSH (28). 2) High level of TGFβ1 inhibits HPG axis directly (29). 3) Chronic disease effect, including the effect of low body weight and malnutrition, inhibits HPG axis and leads to pubertal delay (2, 28, 30). In addition to the mechanisms above, we found that the erythrocyte sedimentation rate, an inflammatory factor, decreased significantly in the follow-up of this patient, while the puberty development was initiated. We thus proposed that severe inflammation may also play a role in the hypogonadotropic hypogonadism in patients with CED, and suppressing the inflammation state may be important for the initiation of puberty development in such patients.

### Treatment Effect of Glucocorticoid and ARB in CED

In this patient, we observed improvement of bone mass in the spine and hip and body fat mass and initiation of puberty development, with the treatment of a small-to-medium dosage of prednisone and losartan. However, no published case has reported the improvement of puberty development using the current treatment (**Table 2**). This may be due to the lack of attention to puberty development or short-term follow-ups in patients with CED.

Glucocorticoid is the most common treatment in CED so far. Glucocorticoid inhibits the activation of TGFβ1 and inflammation. Glucocorticoid also binds to the glucocorticoid receptor in adipocyte and osteoblast, which inhibits osteoblast differentiation and recovers adipogenesis (31, 32). Therefore, we propose that, with the treatment of glucocorticoid, severe inflammation is inhibited, the adipose mass is increased, and puberty development is gradually initiated (**Figure 3B**). Researchers used to have a concern that the long-term use of glucocorticoid may worsen osteoporosis in CED. However, with the 28-month treatment of glucocorticoid in this patient, BMD in the lumbar spine and femoral neck had increased significantly. As Boot et al. reported, BMD increased significantly with higher Tanner stages (25). Therefore, a small dosage of glucocorticoid improved the puberty development and therefore increased the BMD in the lumbar spine and femoral neck in the patient.

ARB has been shown to reduce TGFβ1 signaling in chronic renal disease, cardiomyopathy, Marfan syndrome and congenital muscular dystrophy (33–35). In recent years, ARB was reported to relieve bone pain and improve lean and adipose tissue in two CED cases (6, 7). It was proposed that the direct inhibition effect of ARB in TGFβ signaling took effect in the treatment in patients with CED (6) (**Figure 3B**).

### Advantages and Disadvantages

This study has certain advantages. We firstly reported a long-term follow-up of combination treatment of glucocorticoid and ARB in a patient with CED. Previous studies have concerns about the long-term use of glucocorticoid in worsening the bone mass, but we found that low bone mass of the spine and hip can be improved with the treatment. Previous studies have not reported the outcome of hypogonadotropic hypogonadism, and we observed the development of puberty with the long-term use of a small-to-medium dosage of glucocorticoid and ARB.

There are also disadvantages to this study. This is a single case report, and conclusions should be drawn with caution. As CED is extremely rare, we performed a literature review of case reports to summarize the current treatment strategies for CED.

## Conclusion

Combination use of glucocorticoid and ARB may be an effective treatment for CED, on the improvement of bone health, fat disposition, and hypogonadotropic hypogonadism. Future studies should be performed to observe the effect of this treatment strategy in more patients with CED, especially on the changes of bone mass and puberty development.

## Data Availability Statement

The datasets for this article are not publicly available due to concerns regarding participant/patient anonymity. Requests to access the datasets should be directed to the corresponding author.

## Ethics Statement

The studies involving human participants were reviewed and approved by the ethical review committee of Peking Union Medical College Hospital. The patients/participants provided their written informed consent to participate in this study. Written informed consent was obtained from the individual(s) for the publication of any potentially identifiable images or data included in this article.

## Author Contributions

LC drafted the manuscript and conducted the literature review. QL, WG, WY, XL, WX, and YJ performed clinical evaluation and management for patients. YJ conceived the research and made critical revisions to the manuscript. All authors contributed to the article and approved the submitted version.

## Funding

This project was funded by National Key Research and Development Program of China (YJ, 2018YFA0800801), Bethune Charitable Foundation (YJ, G-X-2019-1107-1; LC, G-X-2020-1107-16), National Natural Science Foundation of China (LC, 82100946), and Non-profit Central Research Institute Fund of Chinese Academy of Medical Sciences (LC, 2017PT32020 and 2018PT32001), CAMS Innovation Fund for Medical Sciences (2021-I2M-1-002, WX), National Key Research and Development Program of China (2021YFC2501700, WX).

## Conflict of Interest

The authors declare that the research was conducted in the absence of any commercial or financial relationships that could be construed as a potential conflict of interest.

## Publisher’s Note

All claims expressed in this article are solely those of the authors and do not necessarily represent those of their affiliated organizations, or those of the publisher, the editors and the reviewers. Any product that may be evaluated in this article, or claim that may be made by its manufacturer, is not guaranteed or endorsed by the publisher.
